# Supplementary material for: Supporting health and social care professionals in serious illness conversations: Development, validation, and preliminary evaluation of an educational booklet
Source: PLoS One. 2024 May 31;19(5):e0304180. doi: 10.1371/journal.pone.0304180 (PMC11142603; doi:10.1371/journal.pone.0304180)
Supplement: S1 Table — (PDF) [file pone.0304180.s001.pdf]

S1 table: Standards for Quality Improvement Reporting Excellence for Education (SQUIRE-EDU)

Dimension	Description	Location in the manuscript
**Title and abstract**		
1. Title	Indicate that the manuscript concerns efforts to improve health professions education systems and learning	Title page<br>Page 1
2. Abstract	Keywords include a focus on education and learning	Pages 1-2
**Introduction: Why did you start?**		
3. Problem description	Description of the nature and significance of the need for change in the local educational system	Page 3
4. Available knowledge	Summary of what is currently known about the problem, including relevant previous studies	Pages 3-4
5. Rationale	Identify the guiding theory (learning, change, implementation, or other) and how it aligns with the need for change in the local educational system	Pages 3-4
6. Specific aims	Purpose of the project and of this report	Page 4
**Methods: What did you do?**		
7. Context	a. Contextual elements for learning (e.g., setting, program, people, resources, social, geopolitical influences) before the intervention(s)<br>b. The interrelationships between the contextual elements and the local educational and healthcare systems before the intervention(s)	Not applicable
8. Intervention(s)	a. Description of the primary interventions and cointerventions (e.g., faculty or tool development)<br>b. Specify how the interprofessional education team (e.g., faculty, staff, patients, and learners) was part of the design of the intervention	Pages 4-7<br>Figure 1
9. Study of the intervention(s)	a. Approach used to understand the impact of the educational intervention(s) on the learner and beyond, such as impact on patients, families, the community, faculty, educational program, or the healthcare system<br>b. Approach to assess the fidelity of and the iterative changes to the planned intervention(s) over time	Page 8
10. Measures	Quantitative and/or qualitative measures chosen to assess the educational processes	Pages 6

	and outcomes on learners, faculty, educational programs, patients, families, healthcare systems, or communities	
11. Analysis	a. Qualitative and quantitative methods used to draw inferences from the data<br>b. Methods for understanding variation within the data, including the effects of time as a variable	Pages 6, 7, 9
12. Ethical considerations	Approaches to address vulnerability of learner participants	Page 5
**Results: What did you find?**		
13. Results	For each educational intervention and cointervention, provide details about iterative modifications based on the assessment of the learning	Page 11<br>Tables 3-4
**Discussion: What does it mean?**		
14. Summary	Connect the findings to the guiding theory (learning, change, implementation, other) used to direct the change in the local educational system	Pages 12-13
15. Interpretation	Include the impact of the intervention(s) on learners, faculty, educational program, patients, families, healthcare systems, or communities	Pages 14-15
16. Limitations	a. Limits to the generalizability of the work<br>b. Factors that might have limited internal validity such as confounding, bias, or imprecision in the design, methods, measurement, or analysis<br>c. Efforts made to minimize and adjust for limitations	Page 15
17. Conclusions	a. Scalability of the work to other learners and contexts<br>b. Lessons learned for clinical practice, education, and policy	Pages 16
**Other information**		
18. Funding	Sources of funding that supported this work. Role, if any, of the funding organization in the design, implementation, interpretation, and reporting	Title page

Note. Authors should consider every SQUIRE and SQUIRE-EDU item, but it may be inappropriate or unnecessary to include every SQUIRE and SQUIRE-EDU element in a particular manuscript. Not all items have an EDU extension. If there is no EDU extension, use the SQUIRE item. If there is an EDU extension, it may be used on its own or in conjunction with the SQUIRE item.

Ogrinc G, Armstrong GE, Dolansky MA, Singh MK, Davies L. SQUIRE-EDU (Standards for QUality Improvement Reporting Excellence in Education): Publication Guidelines for Educational Improvement. Acad Med. 2019;94(10):1461-1470. doi: 10.1097/ACM.0000000000002750.
