# Supplementary material for: Supporting health and social care professionals in serious illness conversations: Development, validation, and preliminary evaluation of an educational booklet
Source: PLoS One. 2024 May 31;19(5):e0304180. doi: 10.1371/journal.pone.0304180 (PMC11142603; doi:10.1371/journal.pone.0304180)
Supplement: S2 Table — (PDF) [file pone.0304180.s002.pdf]

S2 table: Review of the principles or content of the main communication protocols and methods

**Communication protocols and methods**	**Principles or content**
ADAPT	The ADAPT method was designed to provide a talking map in discussing prognosis. This method proposes a series of signposts that may not all be applicable to a particular patient: 1) **A**sk what the patient knows, what they want to know; 2) **D**iscover what info about the future would be useful for the patient; 3) **A**nticipate ambivalence; 4) **P**rovide information in the form the patient wants; 5) **T**rack emotion.
CONNECT	The CONNECT protocol was designed to organize the key aspects of remote communication: **C**ontext; **O**rganization; **N**ear and **N**iceties; **E**motions; **C**ounseling; **T**aking care.
NURSE	The NURSE method illustrates a number of communicative strategies for responding to emotions. The strategies can be used either individually or in combination: 1) **N**aming; 2) **U**nderstanding; 3) **R**especting; 4) **S**upporting; 5) **E**xploring.
PREPARED	The PREPARED method was designed for communicating prognosis and end-of-life issues with adults in the advanced stages of a life-limiting illness and their caregivers. This method has eight components: 1) **P**repare for the discussion; 2) **R**elate to the person; 3) **E**licit patient and caregiver preferences; 4) **P**rovide information tailored to needs of patients and / or their families; 5) **A**cknowledge emotions and concerns; 6) **F**oster realistic hope; 7) **E**ncourage questions; 8) **D**ocument.
REMAP	The REMAP tool was designed to provide guidance in addressing goals of care through a complex conversation. This method has stages but not all could apply to a particular patient: 1) **R**eframe why the status quo isn't working; 2) **E**xpect emotion and empathize; 3) **M**ap the future; 4) **A**lign with the patient's values; 5) **P**lan medical treatments that match patient values; EXTRA - Expect questions about other treatment; EXTRA - Talk about services that would help before introducing hospice.
SPIKES	The SPIKES protocol consists of six steps for disclosing unfavorable information: 1) **S**etting up the context; 2) assessing the patient's **P**erception; 3) obtaining the patient's **I**nvitation; 4) giving **K**nowledge and information to the patient; 5) addressing the patient's **E**motions with empathic responses; and 6) eliciting the patient's collaboration in **S**ummarizing the meeting and developing a **S**trategy or clear plan for the future.
TUVERI	The Tuveri method is similar to SPIKES, but is more practical because it reports examples of questions and sequences of actions can be used in a complementary way to the former. This method involves:<br>• introducing oneself;<br>• considering having other people participate in the meeting;<br>• being clear about the objective of giving the information (diagnosis, prognosis, treatment plan);

	• starting from the point of view of the interlocutor (subjective reality);<br>• preceding the real bad news with a warning sign (e.g., “I’m afraid the situation is a bit more serious”) followed by a pause of silence to observe the other person’s reaction;<br>• giving the next piece of information in small doses;<br>• making the listener’s perception of reality gradually come closer to clinical reality;<br>• using non-technical language and avoiding medical jargon;<br>• recognizing and giving space for the expression of emotions;<br>• giving the opportunity to ask more questions;<br>• checking the degree of understanding of the information provided.
