# Supplementary material for: Supporting health and social care professionals in serious illness conversations: Development, validation, and preliminary evaluation of an educational booklet
Source: PLoS One. 2024 May 31;19(5):e0304180. doi: 10.1371/journal.pone.0304180 (PMC11142603; doi:10.1371/journal.pone.0304180)
Supplement: S3 Table — (PDF) [file pone.0304180.s003.pdf]

S3 table: Characteristics of the participants to the focus groups (n=15)

**Participants to the focus groups (n=15)**	**N**
**Female gender**	9
**Age,** years, mean (range)	50 (25-72)
**Overall working experience,** years, mean (range)	24.6 (3-40)
**Experience in the current service,** year, mean (range)	14.5 (3-30)
**Professional profile**	
Nurse	6
Physician	4
Social worker	2
Psychologist	1
Architect	1
Bioethicist	1
**Job position**	
Management	8
Clinical	6
Retired	1
**Setting of care**	
Medicine	3
Palliative care	3
Nursing home	3
Association of patients, family carers, and volunteering	3
Supportive services*	2
Emergency	1

*Continuity healthcare service (n=1), forensic medicine (n=1)
