# Supplementary material for: Supporting health and social care professionals in serious illness conversations: Development, validation, and preliminary evaluation of an educational booklet
Source: PLoS One. 2024 May 31;19(5):e0304180. doi: 10.1371/journal.pone.0304180 (PMC11142603; doi:10.1371/journal.pone.0304180)
Supplement: S4 Table — (PDF) [file pone.0304180.s004.pdf]

S4 table: Characteristics of the external review board (n=15)

**External review board (n=15)**	**N**
**Female gender**	9
**Age,** years, mean (range)	52 (25-71)
**Overall working experience,** years, mean (range)	26.4 (3-48)
**Experience in the current service,** year, mean (range)	13.8 (3-28)
**Professional profile**	
Physician	7
Nurse	5
Psychologist	2
Social worker	1
**Job position**	
Clinical	11
Management	3
Education	1
