# Supplementary material for: Supporting health and social care professionals in serious illness conversations: Development, validation, and preliminary evaluation of an educational booklet
Source: PLoS One. 2024 May 31;19(5):e0304180. doi: 10.1371/journal.pone.0304180 (PMC11142603; doi:10.1371/journal.pone.0304180)
Supplement: S5 Table — (PDF) [file pone.0304180.s005.pdf]

S5 table: Clarity, coherence, relevance, and readability for each sub-section of the booklet

Sub-sections of the booklet	**Clarity** Mean (SD)*	**Coherence** Mean (SD)*	**Relevance** (I-CVI)	**Readability** (Gulpease Index)§
**Introduction**	3.6 (0.5)	3.7 0.6)	1	44
**Basic communication skills**	3.7 (0.5)	3.9 (0.4)	1	70
**Difficult communication scenarios**				
Unawareness of disease trajectory or prognosis	3.8 (0.4)	3.9 (0.3)	1	56
Turbulent emotions and prolonged silences	3.7 (0.5)	3.9 (0.3)	1	61
Preferences for aggressive care	3.7 (0.5)	3.9 (0.3)	1	50
Ambivalent care preferences	3.8 (0.4)	3.9 (0.3)	0.93	56
Family carers with different care preferences	3.7 (0.5)	3.9 (0.3)	0.93	56
Choosing care when the patient's preferences are unknown	3.7 (0.5)	3.9 (0.4)	0.93	55
Unrealistic expectations	3.7 (0.5)	3.9 (0.3)	1	58
Balancing hope and realism	3.7 (0.5)	3.9 (0.4)	1	57
Remote communication	3.7 (0.5)	3.9 (0.4)	0.93	56
Other complex communication scenarios	3.8 (0.4)	3.9 (0.3)	0.93	57
**Additional resources**				
Running the family conferences	3.8 (0.4)	3.9 (0.3)	0.93	58
Times and manners of communication	3.8 (0.4)	3.9 (0.3)	1	50
Process of communication	3.7 (0.5)	3.9 (0.4)	0.93	66
Communication protocols	3.5 (0.6)	3.8 (0.4)	0.93	62
**Actionable resources**	3.6 (0.5)	3.8 (0.4)	1	71
**Overall**	3.7 (0.5)	3.9 (0.5)	S-CVI/Ave 0.97 S-CVI/UA 0.53	60

I-CVI, Content validity index for individual items;
S-CVI/Ave, Overall booklet CVI averaging calculation method; S-CVI/UA, Overall booklet CVI Universal Agreement; SD, standard deviation.
*On 4-point Likert scale (from 1=not at all to 4=much)
§The index ranges from 0 (lowest readability) to 100 (maximum readability). Scores below 80, 60 and 40 identify texts that are difficult to read for a 5th grade level (primary school level, 6 to 10 years), 8th grade level (junior secondary school level, 11 to 13 years), and 13th grade level (secondary school level, 14 to 18 years)
