# Supplementary material for: Supporting health and social care professionals in serious illness conversations: Development, validation, and preliminary evaluation of an educational booklet
Source: PLoS One. 2024 May 31;19(5):e0304180. doi: 10.1371/journal.pone.0304180 (PMC11142603; doi:10.1371/journal.pone.0304180)
Supplement: S6 Table — (PDF) [file pone.0304180.s006.pdf]

S6 table: Layout and design characteristics of the booklet based on the Baker Able Leaflet Design (BALD) criteria.

**Design Characteristics**	**3 Points**	**2 Points**	**1 Point**	**0 Point**
Lines 50-89 mm long			Yes	No
Separation between lines	> 2.8mm	2.2-2.8mm		<2.2mm
Lines unjustified			Yes	No
Serif typeface		Yes		No
Type size	12 point	10-11 point	9 point	< 9 point
First Line indented			Yes	No
Titles lower case			Yes	No
Italics		0 words	1-3 words	≥ 4 words
Positive advice		Positive		Negative
Headings standout		Yes		No
Numbers all Arabic			Yes	No
Boxed text			0-1Box	> 1 Box
Pictures	Words count not replace	In between	In between	None or Superfluors
Number of colors	4	3	2	1
White space	>40%	30-39%	20-29%	<20%
Paper quality	> 90gsm	75-90gsm		< 75gsm
Score	12	8	5	0
**Overall score***	**25**			

*BALD score covers 16 attributes and ranges from 0 (worst layout and design) to 32 (best layout and design).

Note. Grey cells indicate the characteristics of the booklet.
