# Supplementary figures and images for: Supporting health and social care professionals in serious illness conversations: Development, validation, and preliminary evaluation of an educational booklet
Source: PLoS One. 2024 May 31;19(5):e0304180. doi: 10.1371/journal.pone.0304180 (PMC11142603; doi:10.1371/journal.pone.0304180)

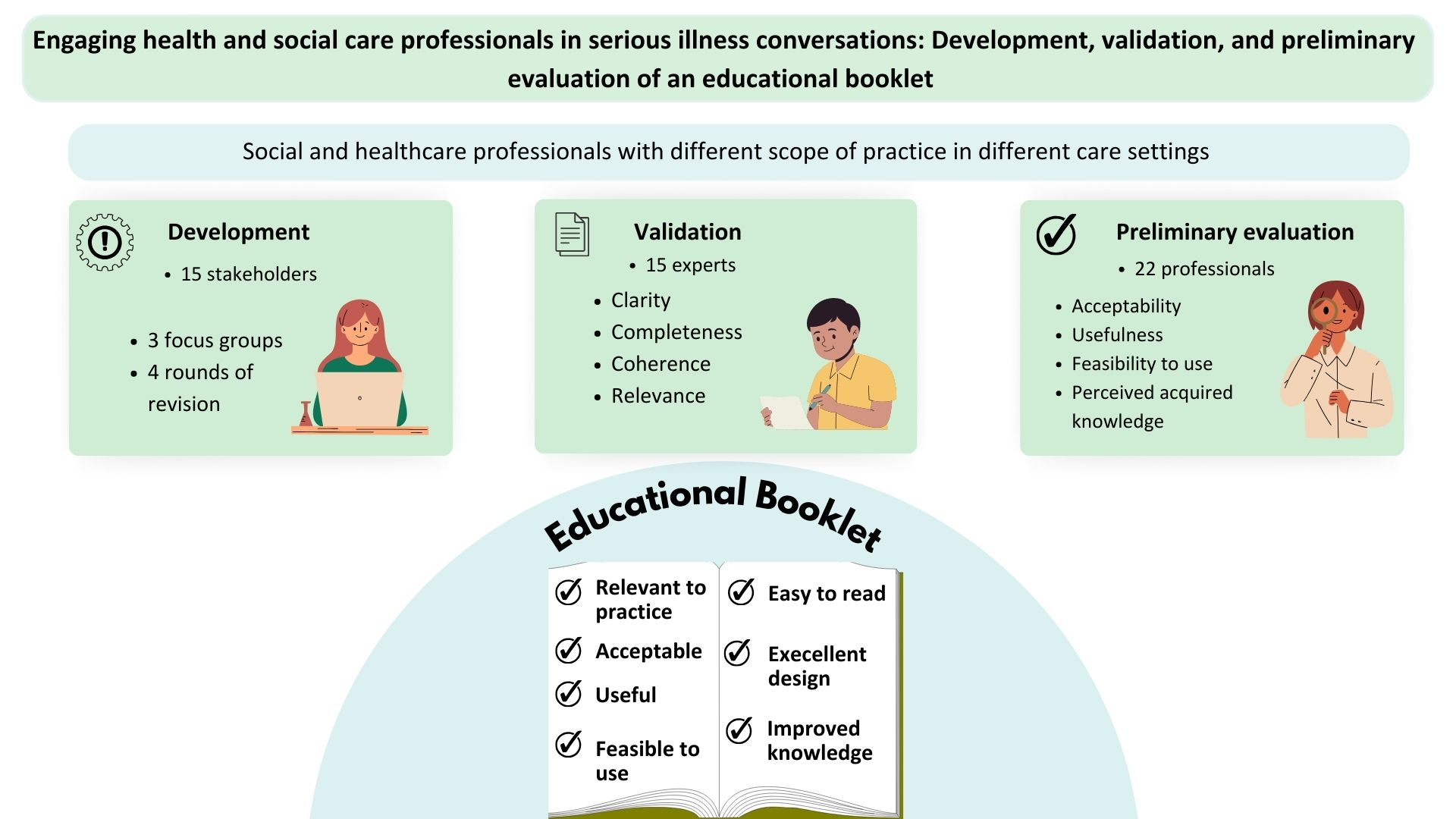

Supplement: S1 Graphical abstract — (TIF) [file pone.0304180.s007.tif]
