# Supplementary material for: Supporting health and social care professionals in serious illness conversations: Development, validation, and preliminary evaluation of an educational booklet
Source: PLoS One. 2024 May 31;19(5):e0304180. doi: 10.1371/journal.pone.0304180 (PMC11142603; doi:10.1371/journal.pone.0304180)
Supplement: Attachment — Submitted filename: Response to Reviewers.docx [file pone.0304180.s008.docx]

| When submitting your revision, we need you to address these additional requirements. | Dear Editor,  Thank you for the opportunity to have our manuscript reviewed. We have uploaded a file labeled 'Revised Manuscript with Track Changes' that shows changes to the points raised by the academic editor and reviewers. Moreover, a copyedited manuscript for language use, spelling and grammar with all changes highlighted has been uploaded as a *supporting information* file. |
| --- | --- |
| 1. Please ensure that your manuscript meets PLOS ONE's style requirements, including those for file naming. The PLOS ONE style templates can be found at  <https://journals.plos.org/plosone/s/file?id=wjVg/PLOSOne_formatting_sample_main_body.pdf> and  <https://journals.plos.org/plosone/s/file?id=ba62/PLOSOne_formatting_sample_title_authors_affiliations.pdf> | We have revised documents according to PLOS ONE’s style requirements. |
| 2. We noticed you have some minor occurrence of overlapping text with the following previous publication(s), which needs to be addressed:  - <https://doi.org/10.3390/healthcare12030398>  In your revision ensure you cite all your sources (including your own works), and quote or rephrase any duplicated text outside the methods section. Further consideration is dependent on these concerns being addressed. | Overlapping text with previous publication has been rephrased as follows:  *The inductive thematic analysis of focus group transcripts identified eight areas for improvement: 1) Make clearer that the booklet is part of a communication skills training program; 2) Strengthen the relational component of communication; 3) Enhance the realism of clinical scenarios; 4) Draw attention to the legal issues; 5) Insert missing critical situations; 6) Employ a person-centered dialogical approach; 7) Enhance actionability; and 8) Improve graphics.*  In the previous publication, such text was reported in the methods section as a result of the primary study, namely the study that is currently under PLOS ONE’s evaluation. Therefore, if the editor(s) agree(s), we would not cite the previous work according to a logic of sequentiality. |
| 3. We suggest you thoroughly copyedit your manuscript for language usage, spelling, and grammar. If you do not know anyone who can help you do this, you may wish to consider employing a professional scientific editing service.  Upon resubmission, please provide the following:  The name of the colleague or the details of the professional service that edited your manuscript  A copy of your manuscript showing your changes by either highlighting them or using track changes (uploaded as a *supporting information* file)  A clean copy of the edited manuscript (uploaded as the new *manuscript* file) | A colleague of ours, Dr. Elena Casabona, copyedited the manuscript for language use, spelling and grammar.  A copy of the manuscript with all changes highlighted has been uploaded as a *supporting information* file and a clean copy of the edited manuscript was uploaded as *manuscript* file, as required. |
| 4. Thank you for stating the following financial disclosure:  “This work was supported by Fondazione Assistenza e Ricerca Oncologica (F.A.R.O.) Onlus. The funding did not influence the study process either the study findings.”  Please state what role the funders took in the study.  If the funders had no role, please state: "The funders had no role in study design, data collection and analysis, decision to publish, or preparation of the manuscript."  If this statement is not correct you must amend it as needed.  Please include this amended Role of Funder statement in your cover letter; we will change the online submission form on your behalf. | We stated the role the funders took in the study, as required.  The following statement has been added:  *The funder had a role in the data collection, but not in study design, data analysis, decision to publish or preparation of the manuscript.* |
| 5. Your ethics statement should only appear in the Methods section of your manuscript. If your ethics statement is written in any section besides the Methods, please delete it from any other section. | The ethics statement now appears only in the Methods section. |
| 6. Please include a separate caption for each figure in your manuscript. | A caption for each figure has been included in the position where the figure should appear. |
| 7. Please include a copy of Tables 1, 2, 3 and 4 which you refer to in your text on page 10 and 11. | All tables have been included in the main manuscript, as required. |
| 8. Please include captions for your Supporting Information files at the end of your manuscript, and update any in-text citations to match accordingly. Please see our Supporting Information guidelines for more information: <http://journals.plos.org/plosone/s/supporting-information>. | Captions for Supporting Information files have been included at the end of the manuscript and the in-text citations updated, as required. |

| Additional Editor Comments:  Please revise according to the reviewer's input   Reviewer #1: Review: Sustaining health and social care professionals in serious illness conversations: Development, validation, and preliminary evaluation of an educational booklet | Thank you for the helpful evaluation of our  manuscript and the constructive suggestions for  improvement. |
| --- | --- |
| Abstract Clarity required about what constitutes a serious illness conversation (i.e. what illness, at what stage of illness trajectory) and whether the patient is an adult or a child. It is not clear from the abstract who (assume healthcare professionals) is aiming to communicate to who (the patient, patient’s family etc) | We have clarified what constitutes a serious illness conversation and who is involved, as follows:  *Serious illness conversations aim to align the care process with the goals and preferences of adult patients suffering from any advanced disease. They represent a challenge for healthcare professionals and require specific skills.* |
| Introduction Please make it clearer much earlier who is communicating to who (as per comments on abstract) | Thank you for the suggestion. We have clarified what it is meant for serious illness conversations and the actors involved, as follows:  *Serious illness conversations represent a wide range of discussions engaging healthcare professionals, patients and/or their informal caregivers when a health condition with a high risk of mortality and a negative impact on quality of life occurs or excessively strains the caregivers [1].* |
| Communication skills training may not be a part of education for healthcare professionals in Italy, but is certainly a growing part of formal professional training for healthcare professionals in other countries. Please acknowledge these differences; the introduction could be strengthened by considering how other healthcare systems attempt to address challenges of communication. | We agree with the reviewer’s suggestion. It’s definitely important to acknowledge differences among countries about different development stages in communication skills training for healthcare professionals. The following paragraph has been added:  *However, there is increasing awareness that communication skills are both innate and teachable [6, 10] and communication skills training is a growing component of formal professional training for healthcare professionals in other healthcare systems such as the UK, US, Canada and Australia, where programs to promote high quality communication have been introduced in the education of healthcare students and also represent a consolidated component of continuing education for professionals [11-14].* |
| The introduction helpfully identifies literature regarding the benefits of written communication guides; these are based on a wide range of different clinical contexts and the section could be strengthened by examination of where these have been used (eg with frail elderly vs acute clinical contexts) and the implications of these different environments for the nature and type of communication. | We have strengthened the introduction by examining the contexts where conversation guides have been used and the impact of the care environment on such tools. The following paragraph has been added:  *Written conversation guides have been introduced in different clinical contexts, such as emergency departments, intensive care units, primary care, oncology, and long-term care to provide high-quality patient-centered care [12, 21, 22]. In these experiences, the original conversation guides were adapted to the care environment and the scope of practice of the healthcare professionals who would use the guide [23].* |
| This section needs a much stronger rationale as to the limitations of the guides which exist already, and why something new is required. | Previous conversation guides provide a talking map that covers the conversation flow with examples of statements that professionals can adopt. However, previous tools do not rely on challenging communication scenarios (e.g., unawareness of disease trajectory or prognosis, ambivalent care preferences) based on clinical case models. This is likely to improve learners’ memory of content because they immediately perceive support for real-case scenarios. Moreover, previous guides employ only one communication strategy. Instead, having more solutions could maximize the chance for learners to find the communication strategy that best fits their learning style (e.g., short tips, infographics, or illustrative statements).  The following paragraph has been added:  *Unlike previous conversation guides [29, 30] that provide a talking map with a series of signposts to cover the conversation flow with no reference to clinical cases, this booklet is built on complex communication scenarios (e.g., unawareness of disease trajectory or prognosis, ambivalent care preferences) based on realistic cases. This is likely to improve learners’ memory of content because they immediately perceive support for real-case scenarios. Moreover, previous guides employ only one communication strategy; instead, having more solutions could maximize the chance for learners to find the communication strategy that best fits their learning style (e.g., short tips, infographics, or illustrative statements).* |
| The paper requires a rationale about why it was decided to use the format of a written communication booklet. Were other modalities considered eg video, audio, online learning etc. | The reviewer is right, in the original manuscript the rationale to use the written format of a communication guide rather than other modalities was missed. The following has been added to solve this criticality:  *Printed educational resources are among the most common approaches to translate research into clinical practice tips [19]. […] Compared to other formats such as video or audio guides, written guides are more quickly accessible in clinical contexts, make it easier to revisit a particular section, and their computerized version allows one to search for keywords that make them more discoverable [19].* |
| Method Please elaborate on the rationale for the clinical cases used and the communication challenges selected to be illustrated in these scenarios. | Challenging communications take place across several clinical settings. To convey this ubiquity, four clinical cases set in contexts where difficult conversations are frequent were developed, including nursing homes, long-term services, the onco-hematological context, and intensive care units.  We made the rationale underpinning the choice of clinical cases explicit as follows:  *“The first draft covered nine difficult communication scenarios against four clinical cases in different contexts of care where difficult conversations are frequent”.*  Communication challenges to be illustrated in these scenarios were selected based on a review of the literature and previous interviews with professionals having varying scopes of practice, as reported at lines 190-192. |
| It would enhance the paper to include a brief review of the communication protocols referenced as examples of ‘good communication scripts’, to include their main principles or content. | Thank you for the suggestion. A brief review of the main communication protocols with a focus on their core principles has been added, as follows:  *All these protocols and methods share core components such as paying attention to the context, exploring patient/family perceptions and preferences and providing information tailored on their needs, and addressing emotions with empathic responses. Each protocol/method also has a specific focus: ADAPT and PREPARED were designed to provide a talking map in discussing prognosis; the NURSE method illustrates communicative strategies for responding to emotions; the REMAP tool may be useful to address goals of care; the CONNECT protocol was designed to organize key aspects of remote communication; finally, SPIKES and TUVERI provide guidance in disclosing unfavorable information.*  Also, a more detailed supplementary table on the content of each communication protocol/methods has been created. |
| Please clarify why representatives from patient associations, carers and volunteers were only drawn from the dementia field, how these representatives were identified and recruited. | We apologise for the inaccuracy. Actually, representatives from patient associations, carers and volunteers were drawn from the field of dementia or palliative care. We reached the reference person of the association –usually the president- that identified their representative. The information has been updated as follows:  *Secondly, 12 health and social care professionals and three representatives of patients associations, family carers, and volunteers in the field of dementia or palliative care were recruited between 5 September 2022 to 26 October 2022 and participated in three focus group discussions (5 participants for each focus group). The research team reached the reference person of each association that then identified their representative.* |
| Please add additional methodological information about the feedback from the focus group participants and how this was integrated into the second iteration of the booklet. It is essential that the methodology for analysing the results of the discussion is clearly described, how potential conflicts of opinion were resolved etc. | We agree with the reviewer on the relevance of transparent methodology to trust the study findings. The following changes have been introduced:  *A member of the research team moderated the discussion using a pre-defined set of questions and solved conflicts of opinion by seeking common ground and areas of agreement to compromise. The questions guiding the focus groups explored the completeness, acceptability, clarity, feasibility to use, and graphics of the booklet, that the literature recognizes as key features for written resources [19]. Another team member took down any field notes on paraverbal and body language. Focus group discussions were audio-recorded, transcribed verbatim and transcripts checked for accuracy. Then, two researchers used an inductive thematic analysis to analyse the transcripts and identify the areas of improvement [37]. Thirdly, this is analysis guided the changes of the second iteration of the booklet, that was then distributed back for a second evaluation with the request to complete a brief open-ended questionnaire assessing its content and design.* |
| Please add the ‘pre-defined set of questions’ as a table or to the body of the manuscript, with a rationale for how these were selected. | The pre-defined set of questions has been added to the body of the manuscript with the rationale for their selection, as follows:  *The questions guiding the focus groups explored the completeness, acceptability, clarity, feasibility to use, and graphics of the booklet, that the literature recognizes as key features for written resources [19].* |
| If the focus groups were not recorded, transcribed and analysed using an established methodology, please state and explain why not. | We confirmed that focus group were audio-recorded, transcribed verbatim, and analysed by employing inductive thematic analysis, as previously mentioned. |
| Validation: please add further detail about the validation process. In particular, further information is required regarding the number of sections which did not achieve an I-CVI of >0.70 and what decisions were made regarding improving these sections or discarding them. Were there differences between the professional raters and those from patient organisations? | As reported in the results section under the paragraph “Second step: Validation” at lines 349-350,  *The final booklet resulted into 17 sub-sections and none was removed based on I-CVI always above 0.9.*  Further details on the I-CVI for each section are reported in the Supplementary table 5.  Thank you for the input on potential differences between the professional raters and association representatives. We specified that no differences emerged, as follows:  *No differences emerged between professional raters and association representatives (lines 350-351).* |
| Further information is required about the sections which were adjusted, detailing what needed to be addressed to improve relevance, clarity or realism and how this was addressed. | According to our perspective, booklet adjustments and changes introduced over the unfolding drafts to improve relevance, clarity or realism represent a finding of this study. Therefore, they have been reported in the results section under the paragraph “First step: Development of the booklet” and particularly in Table 1.  However, we absolutely agree with the reviewer on the need to clarify in the methods section the rationale underpinning the decisions about what to amend. Therefore, we have integrated the information on how data emerging from focus group were analysed and how they contributed to guide the amendments, as required. |
| The statistical analyses used a methodology based on decision aid development and evaluation. Please explore the similarities and differences between a decision aid and a communication booklet. | Thank you for your comment. The following paragraph exploring similarities and differences between decision aids and educational booklets has been added to the section “Statistical analyses”, as required:  *Decision aids and educational booklets share several similarities, including the multidisciplinary approach, evidence-based soundness, and attention to both content and design. However, some differences also occur: decision aids explicitly state the decision that needs to be considered and appear as active tools to guide the decision-making process by providing detailed focus on options and outcomes and helping people to arrive at decisions that reflect their personal values and preferences. Instead, educational booklets represent a source of information but are not focused on decision points due to their broader perspective [45].* |
| I suggest that presenting the method and then results for each of the phases of development would enhance the readability of the manuscript. | We understand the reviewer’s point, however, the suggested modality of presentation may not be consistent with the journal guidelines that require distinct sections for methods and findings.  We tried to help readability by labelling paragraphs across the manuscript consistently. For example, in the methods it is reported: “First step: Development of the booklet”, “Second step: Validation”, and “Third step: Preliminary evaluation”. Again, in the results section it appears sequentially “First step: Development of the booklet”, “Second step: Validation”, and “Third step: Preliminary evaluation”. |
| Results The amendments to the initial draft of the guide is well documented in Table 1. However, this does not include adequate detail about why these adjustments were made (see comment re methodology used to identify and make decisions about what to amend). | The amendments to the initial draft of the booklet have been justified by clarifying the methodology employed to identify the areas of adjustment. Specifically, in the methods section, we reported that an inductive thematic analysis was adopted to analyse the focus group transcripts and pinpointed what needed to be ameliorated.  In the results section under the paragraph “First step: Development of the booklet”, we made clearer that decisions about what to amend were methodologically supported by the inductive thematic analysis of focus group transcripts, as follows:  *The inductive thematic analysis of focus group transcripts identified eight areas for improvement: 1) […]. Further refinements came after re-evaluation by focus group participants and external reviewers, but without major changes.* |
| Discussion The authors highlight the importance of developing a resource to support person-centred conversations; please add in reflection about the decision not to use A Person-Centred Approach methodology. Elements of this methodology have been used by the team, but there is no mention of this within the paper. | We agree with the reviewer’s comment and recognize the adoption of elements of a person-centred methodology, such as focus groups discussions, without explicitly referring to such a methodology. In the revised version, we have made reference to the person-centred methodology and added reflections about the decision to use this approach in the co-creation process of the booklet. The following paragraph has been added:  *The co-creation process for developing the booklet relied on focus groups involving a purposive sample of stakeholders. Focus groups represent a person-centered research method that provides the opportunity to discuss own perspective in a collaborative, participatory setting and could be helpful in obtaining viewpoints that are representative of the majority [46].* |
| A substantial section of the discussion is framed around the 12 domains of behaviour change. This is a useful framework, but it would be more logical and helpful to include this in the introduction; currently it feels like a ‘post hoc’ analysis rather than being core to the development of the booklet. | Thank you for this insightful comment that helped us to improve the consistency of the manuscript. We have included the theoretical understanding of the processes involved in changing healthcare professionals’ behaviors in the introduction. Also, more details of the study findings against the theoretical framework have been presented to facilitate reading and improve consistency. The discussion still presents a shorter section around the 12 domains of behavior change to wrap up and match theory and study findings.  The following paragraph has been added to the introduction:  *Although it is acknowledged that there is no “magic bullet” to change professional practice [25]* *and that the context influences the effectiveness of strategies [26], twelve key theoretical constructs have been identified to explain healthcare professionals’ behavior change [27]. In order to promote behavior change, interventions should enhance (1) knowledge and (2) skills, (3) be consistent with social/professional role and identity, (4) serve as persuasive strategies which influence beliefs about capabilities and (5) lead to changes in beliefs about consequences, (6) stimulate reflection on motivation and goals, (7) influence memory, attention, and decision processes, (8) create a facilitative environmental context that triggers behaviors, evoke (9) emotions and (10) behaviours while providing regulation, and (11) propose desirable behaviours and (12) social norms.*  The following paragraph has been added to the results section:  *Table 4 shows the acceptability score at the item level: particularly, professionals perceived the booklet as consistent with their professional identity and standard by agreeing that it aligns with the way they think things should be done (15/22) and complements their usual approach to communication (14/22). All but three professionals perceived a high probability that using this booklet may result in more benefit than harm, and half believed that the booklet would improve communication and relationships between professionals and families. Around 60% (13/22) reported that they would continue to use the resource after the end of the project.*  The discussion section around the 12 domains of behaviour change has been shortened and reworded, as follows:  *The booklet enhances knowledge to engage successfully in serious illness conversations, provides guidance for real-life difficult communication scenarios while ensuring readability and appealing design the positively influence the memory of the content, is consistent with professional identity and standard, and influences beliefs about capabilities and outcomes with most professionals reporting they would continue to use the resource and perceiving benefits in communication and relationships with families. Additionally, the booklet may stimulate reflection on professional competence and learning needs, enhance sensitivity on the topic of quality and timely communication, which triggers change in behavior, evoke emotions and behaviors related to previous communication experience while providing functional tools for their management, and propose desirable behaviors or instigate a process of social influence.* |
| The decision to involve a design agency is not included in the method an needs earlier integration into the paper, with a clear rationale about the problem being addressed. | Thank you for this comment. The reviewer is right, in the original version of the manuscript we did not provide any information on the decision to involve a design agency and reference to a reference to the involvement of a graphic designer appears late in Table 1 as a result of external criticalities highlighted by the external review board. The research team realised the need to involve a design agency during the development of the booklet due to the recurring design and layout issues emerging over the process, despite the efforts to solve these criticalities already after the first round of feedback (see Table 1 for details).  The following has been added to the methods section under the paragraph “First step: Development of the booklet”:  *The research team initially took the decision not to involve a design agency and to deal on their own with design issues that could have arisen. However, the team later realized that it was not possible to address satisfactorily criticalities about font, colors, and layout as they recurred on the unfolding versions of the booklet despite the efforts to solve the problems; therefore, a graphic designer was involved after the feedback from the external review board.* |
| The discussion briefly mentions ‘the maternal-infant context’. I assume this refers to talking to adults when the patient is an infant or child? Please clarify the language around this. The issues relating to communication about a child’s illness are related but also include separate, additional challenges. This needs much more careful consideration than a single line in the discussion. | We have clarified the language and exploded the issues relating to communication about an infant’s or child’s illness, as follows:  *Moreover, issues related to communication with adults about an infant or child’s illness are not addressed. Parents are often scared, feel guilty, and may have difficulties retaining and processing information. When communicating to parents, professionals have to listen authentically, avoid judgement and act empathically to capture their history, values, perspectives and peculiarities that make one family different from another. Parents need to be supported and accompanied in the experience of their child’s illness, beyond receiving information that should be tailored with regard to content and modalities.* |
| Please expand the section discussing the limitations of the booklet and its development. For example (but not exclusively), would any qualitative work have contributed to the evaluation of the booklet? What are the pros and cons of a physical booklet for wider distribution? How are the authors planning to disseminate the booklet? What impact do they anticipate it would have on patient care and how could this be evaluated? Limitations of the patient scenarios given the broader context of different types of chronic illness? | Thank you for the several input. The limitations have been expanded as follows:  *The booklet presents a limited number of advanced chronic or terminal illnesses, but addresses the most frequent communication scenarios across different clinical settings and offers practical tips and examples of communication protocols that can be tailored to different chronic conditions. Moreover, issues related to communication with adults about an infant or child’s illness are not addressed. Parents are often scared, feel guilty, and may have difficulties retaining and processing information. When communicating to parents, professionals have to listen authentically, avoid judgement and act empathically to capture their history, values, perspectives and peculiarities that make one family different from another. Parents need to be supported and accompanied in the experience of their child’s illness, beyond receiving information that should be tailored with regard to content and modalities. Therefore, a specific resource to support communication in this setting is warranted. Secondly, the involvement of the design agency occurred late in the development process. Its integration from the outset of the project could have eased addressing the feedbacks and translating them into solutions. However, the design agency had received information on the objectives of the booklet and the main critiques emerged during its development, and the research team provided ongoing supervision. Thirdly, the evaluation of the booklet was mainly quantitative in nature, with professionals reporting ratings on acceptability, usefulness, feasibility of use, and acquire knowledge; despite professionals had the opportunity to report open comments and suggest changes, qualitative work could have contributed more deeply to identify nuances that benefit from improvements.*  Moreover, some input including the potential impact of the booklet on patient care and strategies for dissemination were deemed more suitable to the conclusions and introduced as follows:  *The co-creation process yielded an acceptable resource with good readability and excellent design, high content validity, and a high degree of perceived usefulness and acquired knowledge. Also, the time-based solutions favor the use of the resource in daily practice. All these features make the booklet ideally positioned to encourage changes in professional practice and a valid resource to enhance shared decision-making and care planning. In addition, the booklet has good potential for a wider distribution, as it is available on the website of a high-profile Italian society of palliative care and can be downloaded for free. Finally, translation to English and social media campaigns beyond congress presentations are planned to promote dissemination.* |
| The booklet is aimed at discussions with patients, but particularly in the context of frail elderly or patients who are at end of life, what are the implications about communication with other members of the family or carers? | Thank you for arising this point. We recognized that the original version of the manuscript was not clear about the target. Actually, the booklet aimed to provide professionals with guidance in challenging communication scenarios with frail or terminally-patients and/or their families. We have clarified this point by providing a conceptual definition of “serious illness conversations” early in the introduction (lines 90-92). Such conversations represent indeed a wide range of discussions engaging healthcare professionals, patients and/or their informal caregivers. Moreover, at lines 148-150, we have clarified the aim of the booklet as follows:  *[…] we developed an educational booklet aimed at providing professionals with guidance in challenging communication scenarios with frail or terminally-ill patients and/or their families.* |

| Reviewer #2: The introduction was comprehensive and read well. | Thank you for your feedback and words of appreciation. |
| --- | --- |
| Line 113 and contributes to know the patients’ valued priorities and align care with their preferences. | Comma removed. |
| Methodology included creation of the resource, validation and evaluation. | We have added this sentence early in the methods section to provide an overview of the three-step approach this study adopted. |
| Line 194 Fourthly, the booklet was further adjusted according to the emerging comments | Thank you, we have fixed the typo. |
| Could the authors please provide the pre-set questions used in the focus group? | The pre-set questions used in the focus groups have been added to the main text, as follows:  *The questions guiding the focus groups explored the completeness, acceptability, clarity, feasibility to use, and graphics of the booklet, that the literature recognizes as key features for written resources [19].* |
| Results were provided with appropriate statistics. | Thank you. |
| A link to the booklet itself is provided. I would be interested to read an English version, if available. | Unfortunately, the booklet is currently available only in Italian. However, we are planning its English translation to promote dissemination. |
| The option of communication interventions appropriate for 1, 2 and 5 minutes was an excellent concept. | Thank you. |
| The paragraph on page 14 is long and could be divided into 2. | The paragraph has been shortened and reworded as follows:  *The booklet enhances knowledge to engage successfully in serious illness conversations, provides guidance for real-life difficult communication scenarios while ensuring readability and appealing design the positively influence the memory of the content, is consistent with professional identity and standard, and influences beliefs about capabilities and outcomes with most professionals reporting they would continue to use the resource and perceiving benefits in communication and relationships with families. Additionally, the booklet may stimulate reflection on professional competence and learning needs, enhance sensitivity on the topic of quality and timely communication, which triggers change in behavior, evoke emotions and behaviors related to previous communication experience while providing functional tools for their management, and propose desirable behaviors or instigate a process of social influence.* |
| Suggest add 2 - 3 sentences to the conclusion | Three sentences about the potential impact of the booklet on patient care, potentialities for a wider distribution and strategies for dissemination were added to the conclusions, as follows:  *All these features make the booklet ideally positioned to encourage changes in professional practice and a valid resource to enhance shared decision-making and care planning. In addition, the booklet has good potential for a wider distribution, as it is available on the website of a high-profile Italian society of palliative care and can be downloaded for free. Finally, translation to English and social media campaigns beyond congress presentations are planned to promote dissemination.* |
